# Role of Ceramide Synthase 1 in Oral Leukoplakia and Oral Squamous Cell Carcinoma: A Potential Linchpin for Tumorigenesis

**DOI:** 10.7759/cureus.42308

**Published:** 2023-07-22

**Authors:** Sangamithra Surendran, Reshma Poothakulath Krishnan, Pratibha Ramani, Karthikeyan Ramalingam, Selvaraj Jayaraman

**Affiliations:** 1 Oral Pathology and Microbiology, Saveetha Dental College and Hospitals, Saveetha Institute of Medical and Technical Sciences, Saveetha University, Chennai, IND; 2 Centre of Molecular Medicine and Diagnostics (COMManD), Saveetha Dental College and Hospitals, Saveetha Institute of Medical and Technical Sciences, Saveetha University, Chennai, IND

**Keywords:** salivary expression, ceramide, ceramide synthase-1, oral leukoplakia, oral squamous cell carcinoma

## Abstract

Background

Ceramide (CER), known as a "tumor suppressor lipid," plays a crucial role in promoting apoptosis in cancer cells. Ceramide synthase 1 (CERS1), an enzyme responsible for CER synthesis, holds immense importance. Notably, studies have highlighted that reduced levels of CERS1 confer protection to oral squamous cell carcinoma (OSCC) cells against chemotherapeutic agents like cisplatin. However, there is a scarcity of literature exploring the precise role of CERS1 in OSCC. Further investigation is warranted to unravel the intricate relationship of CERS1 in malignant transformation.

Aim

To compare the salivary CERS1 levels in OSCC, oral leukoplakia (OLK), and healthy individuals.

Materials and methods

Salivary samples from 15 healthy individuals, OLK patients, and OSCC patients each were obtained and an enzyme-linked immunosorbent assay (ELISA) (MyBioSource, Inc., San Diego, CA) was performed to evaluate salivary CERS1 enzyme levels. Descriptive statistics and Kruskal-Wallis analysis were done using SPSS v23.0 software (IBM Corp., Armonk, NY).

Results

There was a significant decrease in salivary CERS1 enzyme levels in OSCC (2.08 +/- 0.36 ng/dl) compared to healthy individuals (6.42 +/- 0.42 ng/dl) and OLK patients (4.73 +/- 0.93 ng/dl) (p = 0.05).

Conclusion

In this study, it was found that CERS1 shows a steady decrease in OLK and OSCC. Further cohort studies with larger sample sizes are needed to provide a basis for the role of CERS1 in OLK and its malignant transformation to OSCC.

## Introduction

Oral squamous cell carcinomas (OSCC), which arise from the mucosal epithelium of the oral cavity, account for more than 90% of all oral cancers. Though there are notable regional and environmental risk factor variations, OSCC is one of the most frequently diagnosed cancers in the world, with an annual global incidence of more than 350,000 new cases and 177,000 deaths [1]. A concerning increase is noted in the incidence of OSCC in the younger demographic (45 years old). Oral potentially malignant disorders (OPMDs) are well-known to manifest prior to the development of OSCC. Oral leukoplakia (OLK) is the most common OPMD. OLK was stated as a predominantly white plaque of questionable risk after excluding known diseases or disorders that carry no increased risk for cancer by a meeting supported by the World Health Organization [2]. The average prevalence of OLK is 0.1%, but this may vary from region to region [3]. There is no clear preference for one gender over another [2]. OLK primarily affects people over the age of 30-40 years and is much more common in smokers than in non-smokers [4]. In numerous studies, the calculated annual risk of malignant transformation of OLK ranges from 2% to 3% or even much higher [5]. There are many quantitatively useful predictors of malignant transformation, such as lesion size, clinical subtype, oral subsite, and the presence or absence of epithelial dysplasia, but these are not accurate for use in individual patients [5]. This also applies to the various molecular markers that have been reported as possible predictive markers of malignant transformation in recent decades. The literature is divided on whether the treatment of OLK is effective in preventing malignant transformation [6].

Ceramides (CER) are sphingolipids with a variety of physiological functions [7]. They are composed of sphingosine and fatty acids [8]. CER acts as a key mediator in sphingolipid metabolism and signaling pathways, regulating a wide range of fundamental cellular responses [9]. It is famously known as a "tumor suppressor lipid" because it potently enhances signaling events that drive apoptosis, cell cycle arrest, and autophagy [10]. Endogenous CER elevation can cause a cascade of biological effects, such as apoptosis, cell cycle arrest, differentiation, and autophagy. There are six distinct ceramide synthases (CERS) that make CER [11]. These are enzymes that are predominantly found in the endoplasmic reticulum. The synthesis of endogenous CER with various fatty acid chain lengths is selected differently by each CERS [12]. The C18 type of CER is produced with the help of ceramide synthase 1 (CERS1). Recent research has discovered that overexpression of CERS1 results in impaired cell growth. Similarly, knocking out CERS1 in head and neck squamous cell carcinoma cells reduced apoptosis via repression of caspase-3 and caspase-9 activity [7]. Studies have also found that a decrease of CERS1 in head and neck squamous carcinoma showed resistance to cisplatin, a major chemotherapeutic drug [9].

Although the role of CERS1 in OSCC is quite established, its possible intricate role in tumorigenesis has not been explained. The possibility of the role of CERS1 to start at a premalignant stage has not been discussed or studied. Although the presence of CERS1 in malignant tissue has been studied and analyzed thoroughly, its presence in saliva, an important oral constituent, has not been done yet. Saliva is a versatile component of the oral cavity and contains numerous enzymes that maintain and regulate the oral environment. If the CERS1 enzyme is found in saliva, it could imply that there is a general presence of this enzyme in the oral cavity, as it is not tissue-specific or exclusive to certain diseases. In this study, we analyzed the presence of CERS1 in the saliva of both OLK and OSCC patients. This is a first-of-its-kind study to the best of our knowledge, hence, is done as a pilot phase study.

The aim of the study was to evaluate the expression of salivary CERS1 in patients with healthy oral mucosa, OLK, and OSCC.

## Materials and methods

This is an institutional-based cross-sectional study that has been reviewed by the Institutional Human Ethical Committee, Saveetha Dental College (IHEC/SDC/PhD/OPATH-1954/19/TH-001) and approved by the board. The participation was purely voluntary and informed consent was obtained from all participants. The participants had the choice to quit the study at any point. The sample size was calculated using G*Power 3.1 software based on previous studies [13]. The sample population was selected randomly from the outpatient department of Saveetha Dental College and Hospitals. The study sample included a collection of stimulated saliva from 45 patients: 15 from patients with OSCC, 15 from patients with OLK, and 15 from healthy individuals. The inclusion criteria consisted of patients with histopathologically confirmed cases of OSCC, OLK, and apparently healthy individuals. Exclusion criteria consisted of patients with any other oral potentially malignant lesions such as oral submucous fibrosis, erythroplakia, or lichen planus.

**Figure 1:**
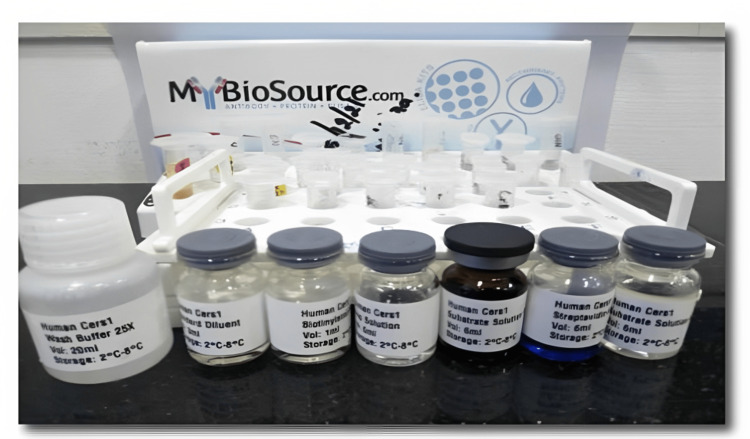
The enzyme-linked immunosorbent assay (ELISA) kit of ceramide synthase 1 (CERS1) enzyme (MyBioSource, Inc., San Diego, CA)

A saliva collection tube was used to collect stimulated saliva. The total amount collected was calculated, excluding the foam. After collecting the samples, they were immediately frozen at -20°C in a deep freezer (Sub-Zero SZ-034, Madison, WI) until processing. The samples were then thawed and centrifuged at 500 g for 10 minutes at 4°C (Neuation iFuge UC02R Universal Refrigerated Genius Centrifuge, Neuation Technologies Pvt. Ltd, Gandhinagar, India). The salivary CERS1 enzyme levels were determined using the enzyme-linked immunosorbent assay (ELISA) (MyBioSource, Inc., San Diego, CA) (Figure 1).

**Figure 2:**
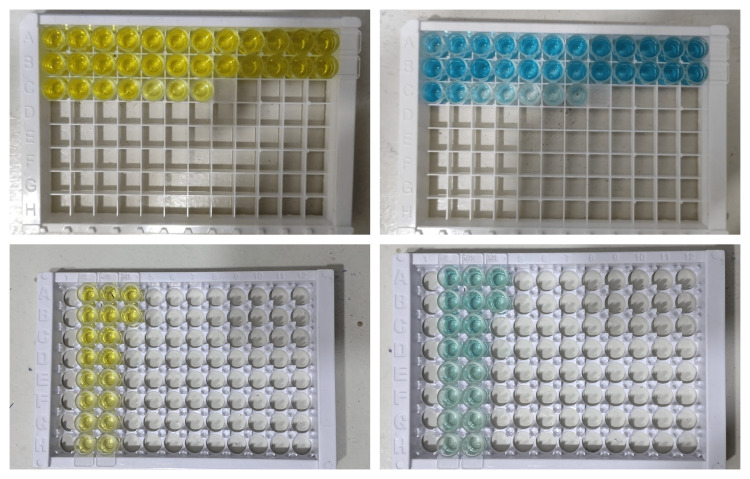
The image shows the change in color of the substrate from yellow to blue due to the antigen-antibody reaction

Before using any reagents, they should be brought to room temperature for at least 30 minutes. If crystals form in the concentrate, gently mix until they dissolve completely. Biotin-antibody 200 l was added to the serum and plasma samples to dilute them and incubated at 37°C for one hour. After that, 90 l of 3,3′,5,5′-tetramethylbenzidine (TMB) substrate was added and incubated for 10-30 minutes. Finally, the optical density due to the change in color from yellow to blue of each well was determined in 30 minutes (Figure 2).

**Figure 3:**
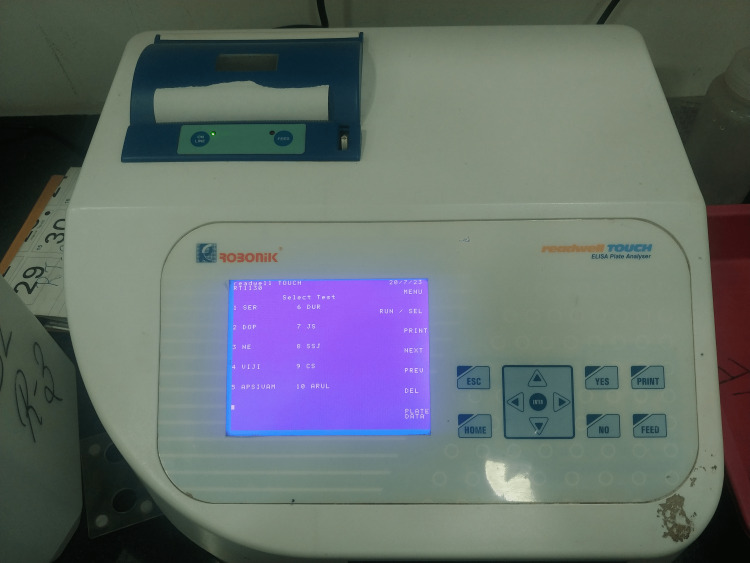
Image showing the microplate reader (Readwell TOUCH ELISA Plate Analyzer, Robonik India Pvt Ltd, Thane, India)

This was done using a 450 nm microplate reader (Readwell TOUCH ELISA Plate Analyzer, Robonik India Pvt Ltd, Thane, India) (Figure 3).

Data are expressed as mean ± standard deviation. Descriptive statistics and Kruskal-Wallis analysis were done using SPSS v23.0 (IBM Corp., Armonk, NY).

## Results

This study was performed to analyze the CERS1 levels among patients with OLK and OSCC in comparison with apparently healthy individuals. The study samples included 23 males and 22 females. The mean age of the included patients was 45.5 years. The study showed the presence of CERS1 in the saliva of all the included subjects by evaluating the change in color of the substrate from blue to yellow (Table 1).

**Table 1:** Descriptive statistics Table showing the descriptive statistics of salivary CERS1 levels in oral squamous cell carcinoma, oral leukoplakia, and healthy individuals. OSCC: oral squamous cell carcinoma; CERS1: ceramide synthase 1.

	N	Minimum	Maximum	Mean	Std. deviation
Normal	15	5.64	6.89	6.428	0.42012
Leukoplakia	15	2.63	5.45	4.7233	0.93
OSCC	15	1.37	2.5	2.084	0.36734

**Figure 4:**
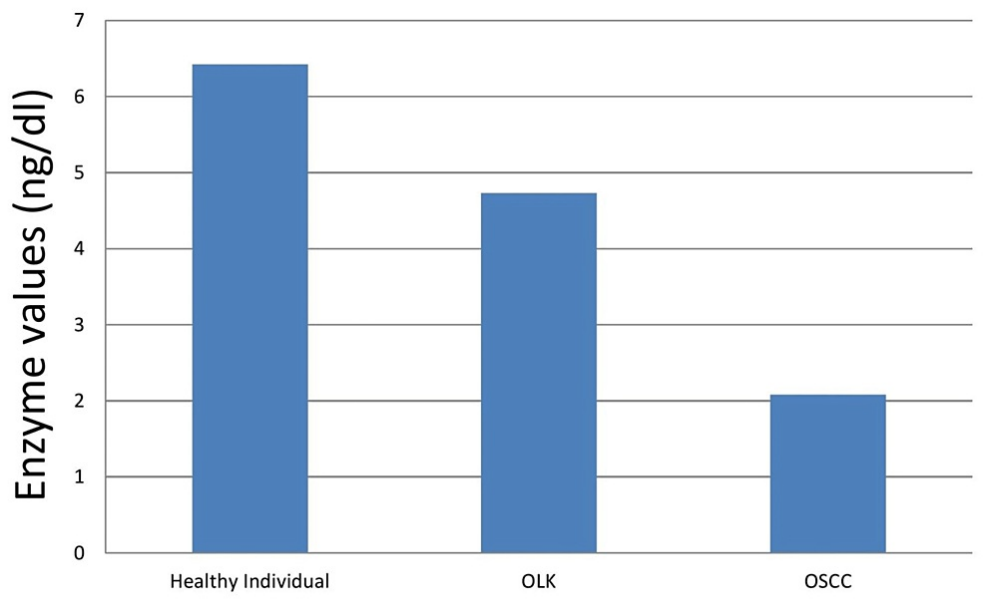
Salivary CERS1 levels The graph shows the enzyme levels of salivary ceramide synthase 1 (CERS1) in oral squamous cell carcinoma (OSCC), oral leukoplakia (OLK), and healthy individuals. The graph shows a steady decrease in CERS1 levels in OLK and OSCC.

There was a significant decrease in the salivary CERS1 level in OSCC patients (2.08 +/- 0.36 ng/dl) compared to healthy individuals (6.42 +/- 0.42 ng/dl). There was a difference of 4.34 ng/dl between healthy individuals and OSCC patients (p = 0.05). There was also a significant decrease in salivary CERS1 level in OSCC patients (2.08 +/- 0.36 ng/dl) compared to OLK patients (4.73 +/- 0.93 ng/dl) (p = 0.05) (Figure 4).

There was a difference of 2.29 ng/dl between OLK and OSCC patients (p = 0.05). There was a decrease in the salivary CERS1 in OLK patients (4.73 +/- 0.93 ng/dl) compared to healthy individuals (6.42 +/- 0.42 ng/dl), but the results were not significant. This had a difference of 1.69 ng/dl (p > 0.05). Overall, there is a steady decrease in salivary CERS1 levels as the disease progresses (Table 2).

**Table 2:** Salivary CERS1 level in oral squamous cell carcinoma, oral leukoplakia, and healthy individuals The table shows salivary CERS1 levels in oral squamous cell carcinoma, oral leukoplakia, and healthy individuals. Kruskal-Wallis analysis shows a significant decrease in oral squamous cell carcinoma compared to healthy individuals. OSCC: oral squamous cell carcinoma; CERS1: ceramide synthase 1.

	Normal	N	Mean rank	P-value (Kruskal-Wallis test)
Leukoplakia	9	7	4.6	0.08
	6	8	6.4	
	Total	15		
OSCC	9	7	2.8	0.05*
	6	8	6.2	
	Total	15		

## Discussion

CERS1 is an enzyme that catalyzes the production of ceramide, a sphingosine-family lipid molecule that has been found to be a potent antitumor molecule [14]. In this study, we explored the role of CERS1 in OSCC and OLK. This is a first-of-its-kind study, wherein we aimed to find the presence of CERS1 in the saliva of patients with OSCC and OLK. We also aimed to find the difference in salivary CERS1 levels in OSCC patients, OLK patients, and healthy individuals. The study showed the presence of CERS1 in the saliva of all the included subjects. This proves that CERS1 plays a role in maintaining the oral microenvironment. CERS1 is an enzyme that catalyzes the synthesis of C18 ceramide in a fumonisin B1-independent manner [15]. CERS1 is found within the cell in the endoplasmic reticulum and the Golgi apparatus membrane [16]. In the present study, we found that there was a significant decrease in the salivary CERS1 level in OSCC patients (2.08 +/- 0.36) compared to healthy individuals (6.42 +/- 0.42), with a mean difference of 4.34 (p < 0.05). This value is consistent with the previous results, where tissue CERS1 levels were compared between OSCC patients and healthy individuals. CERS1 makes use of C18-acyl-CoA, which is linked to autophagic cell death in oral cancer cells [17]. CERS1 is found to activate the mTOR/P13 pathway. CER inhibits protein kinase B activity by activating protein phosphatase 2A, p3844, and protein kinase, and then AKT inhibits BCL2 phosphorylation. Finally, a decrease in BCL2 levels and the BCL2 to BAX ratio results in cell death. This effect was confirmed by immunohistochemical studies [18]. CERS1 inhibition inhibits apoptosis by inhibiting the synthesis of C18 CER, AKT, and BCL2.

Mitophagy is a significant concept in the CERS1 with head and neck squamous cell carcinomas (HNSCC). Mitophagy can play a more significant part in tumor suppression when lethal mitophagy, which is the process by which mitochondria are degraded to the point where the cell dies without undergoing apoptosis, is activated [19]. Apoptosis occurs through either simple upregulation of mitophagy or sustained mitophagy for long periods of time via the leakage of cathepsin proteases from the lysosome [20]. CERS1 and its byproduct C18-ceramide play a role in lethal mitophagy but independently of caspase 3, Bax, and Bak. Lethal mitophagy has been shown to inhibit HNSCC both in vitro and in vivo [21]. It was found that C18-ceramide-mediated lethal mitophagy in HNSCC was distinct from cell starvation-induced survival autophagy. Mitophagy may be activated and regulated by cancer cells via a variety of pathways. It has been discovered that inhibiting the ceramide transfer protein (CERT), which moves ceramide from the endoplasmic reticulum to the Golgi, results in hexosylceramide building up on mitochondria, an increase in reactive oxygen species, an increase in mitophagy, and premature cellular senescence [22].

Our study found a significant decrease in salivary CERS1 levels in OSCC patients compared to OLK patients with a mean difference of 2.29. Studies have found the presence of CER in pharyngeal leukoplakia and the results are consistent with the present study [23]. CERS1 is found to activate endoplasmic reticulum stress. Endoplasmic reticulum stress triggers an intrinsic apoptosis pathway that includes mitochondrial Bax translocation, cytochrome C release, and caspase 3 cleavage [24]. Unrepaired damage triggers the action of p53, Bax, p38, JNK, and stress-activated protein kinases (SAPKs), which stop proliferation and trigger apoptosis [25]. Contrarily, SAPK functions independently of p53 and controls apoptosis through a novel mechanism that may downstream caspases [26,27]. Along with SAPK and cell cycle checkpoints, members of the Bcl-2 family that act downstream of p53 can also cause apoptosis. Similar research on gastric cancer revealed that Bak overexpression caused the cells to undergo apoptosis. These prove that there is a possible role of CERS1 in the tumorigenesis of OLK to OSCC. Further studies with more inclusion criteria are needed for a more concrete result.

Based on the results obtained, we have proposed a pathway expressing the role of salivary CERS1 in OLK and its possible tumorigenesis into OSCC. Unrepaired damage releases reactive oxygen species, which induce hypoxia in the cells. This results in endoplasmic reticulum stress. Here, due to the stress, there is a decrease in CERS1 production, which causes decreased CER formation. This causes an increase in sphingosine-1-phosphate (S1P), which activates two pathways. One, there is an increase in Bcl2, which causes an increase in caspase 2 and 9, which causes decreased apoptosis and increased cell proliferation. Second, there is an increase in vascular endothelial growth factor (VEGF), which causes angiogenesis. This leads to increased invasion and metastasis [28].

As was previously mentioned, pro-survival S1P production has increased while pro-apoptotic C18-ceramide production has decreased in many cancers [29]. By blocking CERS1 or upregulating ceramide-metabolizing enzymes, cellular C18-ceramide levels can be decreased [28]. Therefore, radiotherapy and chemotherapy work to sensitize cancer cells and trigger apoptosis by increasing ceramide levels. Numerous studies support this finding. Serum C18-ceramide levels were elevated in some patients participating in a phase II clinical trial receiving gemcitabine and doxorubicin for head and neck cancer. In comparison to patients with normal serum C18-ceramide levels (progressing disease state), these patients had better clinical outcomes (partial response, complete response, or stable disease state) [30]. These results were confirmed in mouse xenograft tumors and HNSCC cell lines, which demonstrated a CERS1-dependent rise in C18-ceramide generation following treatment. Even substances that resemble ceramide structurally have been demonstrated to inhibit cell proliferation by inducing mitophagy, as was the case with native solenopsin treatment of melanoma cell lines [13,31]. Cancer cells can convert ceramide to other sphingolipids, specifically in an effort to avoid apoptosis or lethal mitophagy and lessen the effectiveness of the treatment. Ceramide can be hydrolyzed by ceramidases and transformed into the pro-survival compound S1P, or it can be glycosylated by the enzyme glucosylceramide synthase and transformed into the compound glucosylceramide, which is associated with drug resistance to chemotherapeutics [23]. Using lysosomal inhibitors to stop cancer's protective mitophagy made drug-resistant HNSCC cells more susceptible to therapeutic ceramide delivery via C6-ceramide nanoliposomes [32]. In contrast, neutral ceramidase inhibition increased the production of ceramide and autophagy, which protected against drug-induced necroptosis in mouse embryonic fibroblasts [32].

Limitations

Our study has identified salivary CERS1 values in OLK and OSCC patients. But, we had not segregated the samples based on the histological severity of epithelial dysplasia or the histological grade of OSCC. The patients were not screened for habits. Larger multi-centric studies with the inclusion of multiple OPMDs will provide crucial information regarding the role of CERS1 in the pathogenesis of OSCC.

## Conclusions

Ceramide metabolism is being investigated further in light of its role in cancer survival, death, and resistance to chemotherapeutics and immunotherapies. Understanding the way ceramide and CERS1 sensitize or desensitize cancer is essential for developing new cancer therapies for patients. Ceramide's subcellular location on mitochondria has been shown to induce a potent anticancer effect by inducing lethal mitophagy. More research is needed to fully understand the intricate relationships that these ceramides have in controlling tumor growth and interacting with the immune system.
